# Statistical Analysis of Efficient Unbalanced Factorial Designs for Two-Color Microarray Experiments

**DOI:** 10.1155/2008/584360

**Published:** 2008-06-18

**Authors:** Robert J. Tempelman

**Affiliations:** Department of Animal Science, College of Agriculture and Natural Resources, Michigan State University, East Lansing, MI 48824-1225, USA

## Abstract

Experimental designs that efficiently embed a fixed effects treatment structure within a random effects design structure typically require a mixed-model approach to data analyses. Although mixed model software tailored for the analysis of two-color microarray data is increasingly available, much of this software is generally not capable of correctly analyzing the elaborate incomplete block designs that are being increasingly proposed and used for factorial treatment structures. That is, optimized designs are generally unbalanced as it pertains to various treatment comparisons, with different specifications of experimental variability often required for different treatment factors. This paper uses a publicly available microarray dataset, as based upon an efficient experimental design, to demonstrate a proper mixed model analysis of a typical unbalanced factorial design characterized by incomplete blocks and hierarchical levels of variability.

## 1. INTRODUCTION

The choice and optimization of experimental designs for two-color microarrays have been receiving increasing attention [1–13]. Interest has been particularly directed towards optimizing experiments that involve a factorial design construction [7, 9, 14] in order to study the joint effects of several factors such as, for example, genotypes, pathogens, and herbicides. It is well known by plant scientists that factorial designs are more efficient than one-factor-at-a-time studies and allow the investigation of potentially interesting interactions between two or more factors. For example, investigators may study how herbicide effects (i.e., mean differences) depend upon plant genotypes or times after application.

Two-color systems such as spotted cDNA or long oligonucleotide microarrays involve hybridizations of two different mRNA samples to the same microarray, each of the two samples being labeled with a different dye (e.g., Cy3 or Cy5; Alexa555 or Alexa647). These microarrays, also simply referred to as arrays or slides, generally contain thousands of probes with generally a few (≤4) spots per probe, and most often just one spot per probe. Each probe specifically hybridizes to a matching mRNA transcript of interest within each sample. After hybridization, microarray images are scanned at two different wavelengths as appropriate for each dye, thereby providing two different fluorescence intensity measurements for each probe. Upon further preprocessing or normalization [15], these dye-specific intensities for each probe are believed to reflect the relative mRNA abundance for the corresponding transcript within the respectively labeled samples. The normalized intensities, or the Cy3/Cy5 ratio thereof, for each spot are typically logarithmically transformed to render data that is generally characterized to be approximately normally distributed.

An increasingly unifying and indisputable message is that the heavily used common reference design is statistically inefficient [1, 9, 10, 12, 13]. Here, the same common reference sample or pool is reused as one of the two samples on every microarray, the other sample deriving from a treatment group of interest. Hence, inferences on differential expression are based only on indirect connections across arrays as samples from different treatments of interest are never directly connected or hybridized together on the same microarray. In contrast, most of the alternatively proposed efficient designs are incomplete block designs, the most popular being various deviations of the loop design as first proposed for microarrays by Kerr and Churchill [16]. In these designs, direct connections or hybridizations are typically reserved for the most important treatment comparisons with inference on other comparisons being generally as efficient as any based on the common reference design.

The intent of this review is to reemphasize the use of mixed models as the foundation for statistical analysis of efficient factorial designs for microarrays. Mixed model analysis for microarray data was first proposed by Wolfinger et al. [17]. However, this and other previous expositions on the use of mixed model analysis for microarray data have been primarily directed towards the analysis of completely balanced designs [18, 19] whereas many recently proposed designs for microarray studies are unbalanced with respect to, for example, different standard errors on all pairwise comparisons between treatment groups [10, 13]. We will review various aspects of mixed model analysis for unbalanced designs, including a demonstration on publicly available data from a recent plant genomics study [20].

## 2. THE CONNECTION BETWEEN MIXED MODELS AND EFFICIENT DESIGNS

Efficient experimental designs are typically constructed such that their factors can be broadly partitioned into two categories: *treatment structure* factors and *design structure* factors [21]. The treatment structure naturally includes the factors of greatest interest; for example, herbicides, genotypes, tissues, and so forth, whose effects are deemed to be fixed. In other words, the levels of these *fixed effects* factors are specifically chosen by the investigator such that mean comparisons between such levels, for example, different treatments, are of primary interest. These factors also include any of whose levels are consistently reused over different experiments, such as dye labels, for example, Cy3 versus Cy5, for two-color microarrays. On the other hand, the design structure primarily includes *random effects* factors, whereby the levels of each such factor are considered to be randomly chosen from a conceptually infinite set of such levels [22]. For example, the specific arrays used for a microarray study are considered to be a random sample from a large, perhaps hypothetically infinite, population of arrays; similar claims would be made regarding biological replicates, for example, plants, pools thereof, or even field plots as dependent upon the experimental design [14]. Within each random-effects factor, the effects are typically specified to be normally, independently, and identically distributed (NIID) with variability in effects formally quantified by a variance component (VC).

These design structure or random effects factors are typically further partitioned into two subcategories: *blocking* factors and *experimental error* factors. In two-color microarray experiments, arrays are typically blocking factors as treatments can be directly compared within arrays, although this is not true for the common reference design as previously noted. Blocking represents a longstanding and efficient experimental design strategy for improving precision of inference on treatment comparisons. Experimental error factors, such as plants or pooled samples thereof within treatments, are often necessary to be included as random effects in order to properly specify true experimental replication at the biological level rather than merely at the measurement or technical level. Such specifications are particularly required when multiple aliquots are derived from the same biological specimen for use in multiple arrays [20, 23] or when probes for each gene transcript are spotted more than once on each array. Of course, plants may also alternatively serve as blocking factors in some designs if different tissues are compared within plants.

Currently, there is much software available for microarray data analysis, some of which is only suited for studies having only a treatment structure but no pure design structure. Common examples include the analysis of data generated from single channel systems (e.g., Affymetrix) or of log ratios generated from common reference designs. When no random effects are specified, other than the residuals, the corresponding statistical models are then simply fixed-effects models. Ordinary least squares (OLS) inference is then typically used to infer upon the treatment effects in these studies. OLS is appropriate if the assumption is valid that there is only one composite residual source of variability such that the residuals unique to each observation are NIID.

Conversely, statistical analysis of efficient two-color experiments having a fully integrated treatment and design structure needs to account for fixed and random effects as typical of a *mixed effects* model, more often simply referred to as a mixed model. Generalized least squares (GLS) analysis, also referred to as mixed-model analysis, has been recognized as optimal in terms of minimizing variance of estimates for inference on treatment comparisons. This is true not only for efficient microarray designs [10, 17, 19, 24] but even for general plant science and agronomy research [25–27], including recent applications in plant genomics research [20, 23, 28]. Some of the more recently popular microarray data analysis software has some mixed model analysis capabilities [29, 30].

**Figure 1:**
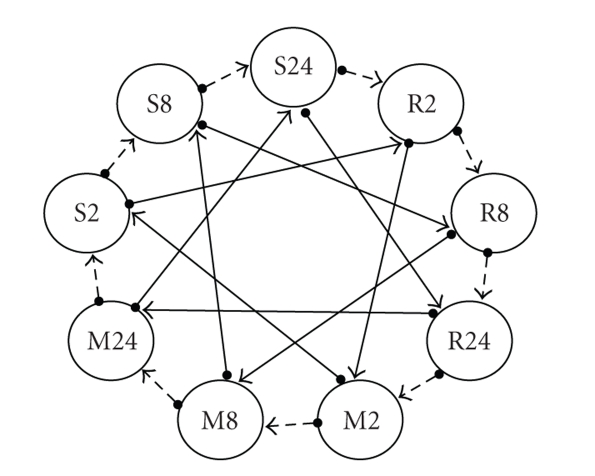
Optimized interwoven loop design for 9 treatments using R package SMIDA (Wit et al., 2005). Each circle represents a different treatment group. Each arrow represents a single array hybridization with circle base representing the Cy3 labeled sample and tail end representing the Cy5 labeled sample.

Recall that some designs may be characterized by different levels of variability thereby requiring particular care in order to properly separate biological from technical replication, for example. Hence, it is imperative for the data analyst to know how to correctly construct the hypothesis test statistics, including the determination or, in some cases, the estimation of the appropriate degrees of freedom. Although, some of these issues have been discussed for balanced designs by Rosa et al. [19], they have not generally been carefully addressed for the analysis of microarray data generated from unbalanced designs. Optimally constructed experimental designs are often unbalanced with respect to inference on all pairwise treatment comparisons, such that even greater care for statistical inference is required than in completely balanced designs. For example, Wit et al. [13] proposed a method for optimizing two-color microarray designs to compare any number of treatment groups. Suppose that 9 different treatment groups are to be compared. Using the methods and software developed by Wit et al. [13], the recommended interwoven loop design that is optimized for A-optimality (lowest average squared standard errors for a particular arrangement of treatment comparisons) is provided in Figure 1. Although Figure 1 appears to be visually symmetric with respect to the treatment labels, including that all treatment groups are dye balanced, not all treatment groups are directly hybridized against each other. Hence, inferences on all pairwise comparisons between treatment groups will not be equally precise. For example, the standard errors for the inference on treatments R2 versus R8 or R8 versus R24 will not be the same as that for treatments R8 versus S24 or R8 versus M2 due to the differences in the number and/or degree of direct and indirect connections for these two sets of comparisons in Figure 1.

Even for some balanced factorial designs, where the standard errors for comparing mean differences for levels of a certain factor are the same for all pairwise comparisons, the experimental error structure can vary substantially for different factors. That is, substantial care is required in deriving the correct test statistics, particularly with split plot arrangements [14]. Of course, even when a completely balanced design is intended, data editing procedures that delete poor quality spots for certain genes would naturally result in unbalanced designs.

## 3. CASE STUDY

### 3.1. Design

Zou et al. [20] present an experiment where three different inoculate treatments were applied to soybean (*Glycine max.*) plants 14 days after planting. The three different inoculates included bacteria inoculation along with the avirulence gene *avrB* thereby conferring resistance (R), bacteria inoculation without *avrB* thereby conferring susceptibility (S), and a control group whereby the inoculate simply contained an MgCl_2_ solution (M). Unfoliated leaves from three to four plants were drawn and pooled for each treatment at each of three different times after postinoculation; 2, 8, and 24 hours. Hence, the treatment structure was comprised of a 3 × 3 factorial, that is, 3 inoculates ×3 times, for a total of 9 groups. A 10th group involving a fourth null inoculate with leaves harvested at 2 hours postinoculation, N2, was additionally studied by Zou et al. [20]. The complete dataset on gene expression data for all 27 684 genes represented on a set of three microarray platforms as used by Zou et al. [20] is available as accession number GSE 2961 from the NCBI gene expression omnibus (GEO) repository (http://www.ncbi.nlm.nih.gov/geo/). The vast majority of the corresponding probes were spotted only once per array or slide for each platform.

**Figure 2:**
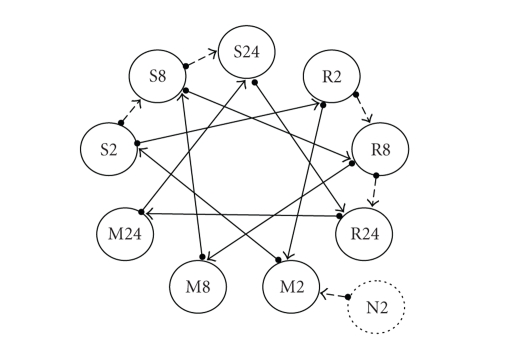
Experimental design for one replicate from Zou et al. (2005). Treatments included a full 3 × 3 factorial of inoculate and time effects plus a 10th null control group at time 2 (N2). Samples indicated by circles with letters indicating inoculate assignment: bacteria resistant (R), a bacteria susceptible (S), and MgCl_2_ (M) control inoculate and numbers indicating time (2, 8, or 24 hours) after inoculation. Each arrow represents a single array hybridization with circle base representing the Cy3 labeled sample and tail end representing the Cy5 labeled sample. Solid arrows refer to the A-loop design of Landgrebe et al. (2006).

A graphical depiction of the 13 hybridizations that superimposes the design structure upon one replicate of the 3 × 3 factorial treatment structure plus the additional 14th hybridization involving the 10th group N2 is illustrated in Figure 2. Note that at least two aliquots per each pooled sample are used, each aliquot being labeled with different dyes such that each replicate pool is used in at least two different hybridizations or arrays with opposite dye assignments. In other words, this design is characterized by technical replication such that it is imperative to explicitly model samples within inoculate by time combination as the biological replicates, that is, a set of random effects for modeling experimental error. Failing to do so would confuse pseudoreplication with true replication in the statistical analysis as each of the 2+ aliquots per each pool would then be incorrectly counted as 2+ different experimental replicates. The design in Figure 2 was replicated twice by Zou et al. [20], the second replication being of the exact same dye assignment and hybridization orientation as the first, for a total of 28 hybridizations. Hence, there were 20 samples (pools of leaves) utilized in the experiment, 2 per each of the 9 inoculate by time treatment groups plus 2 samples for the N2 control.

We arbitrarily consider gene expression measurements for just one particular gene based on the GEO submission from Zou et al. [20]: ID_REF #30 located in the metarow-metacolumn-row-column location 1-1-2-14 of each array from microarray platform GPL 1013, one of three different platforms used by Zou et al. [20] and further described in GEO. The statistical analysis of any of the remaining 27 683 genes that were spotted once on each slide across the three different platforms would be exactly the same as that for ID_REF #30, at least for those genes where no observations would be edited out for poor data quality. We use the normalized Cy3 and Cy5 data, provided as fields *S532N* and *S635N* in accession number GSE 2961 for ID_REF #30 from GEO. Hence, for the 28 hybridizations considered for two replications of Figure 2, there were 56 fluorescence intensities (28 Cy3 and 28 Cy5) for each gene. The 56 fluorescence intensities for ID_REF #30, as retrieved from GSE 2961 in GEO, are reproduced in Table 4 .

### 3.2. Statistical model

For the purposes of this review, we concentrate our attention just on the subdesign characterized by the solid arrows in Figure 2 that connect the three primary inoculates (R,S, and M) together within each of the 3 different times (2, 8, and 24 hours). The remaining dashed lines in Figure 2 involve either the 10th group (N2) or connect adjacent times (2 with 8 and 8 with 24) within each of two inoculates (R and S); note that no hybridizations connecting any of the three times within inoculate M were provided with GSE 2961 on GEO. Labeling inoculate type as Factor A and time after inoculation as Factor B, the resulting subdesign is an example of the “A-loop” design presented by Landgrebe et al. [9] as illustrated in their Figure 2 (B), albeit for a 3 × 2 factorial treatment structure in their case. In other words, the only direct connections between the 9 treatment groups within arrays involve comparisons of levels of Factor A within levels of Factor B. Using the log intensities as the response variables for further statistical analysis, an appropriate linear mixed model to specify for this A-loop design would be as follows:

(1) $$y_{ijklm}=\mu +\alpha_{i}+\beta_{j}+\alpha \beta_{ij}+\delta_{k}+r{\left(\alpha \beta \right)}_{l:ij}+s{\left(\beta \right)}_{m:j}+e_{ijklm},$$

where *y*_*ijklm*_ is the log fluorescence intensity pertaining to the *l*th biological replicate assigned to the *i*th inoculate (*i* = 1, 2, 3) and *j*th time (*j* = 1, 2, 3) labeled with the *k*th dye (*k* = 1 or 2), and hybridized to array *m*(*m* = 1, 2,…, 6) within the *j*th time. Here, *μ* is the overall mean, *α*_*i*_ is the effect of the *i*th inoculate, *β*_*j*_ is the effect of the *j*th time, *α* *β*_*ij*_ is the interaction effect between the *i*th inoculate and *j*th time, and *δ*_*k*_ is the effect of the *k*th dye, all of which are defined to be fixed effects. The design structure component of (1) is defined by the random effects of *r*(*α* *β*)_*l*:*ij*_ for the *l*th pool or biological replicate (*l* = 1, 2) within the *ij*th inoculate-time combination, *s*(*β*)_*m*:*j*_ for the *m*th array (*m* = 1, 2,…, 6) or slide within the *j*th time, and the residual *e*_*ijklm*_ unique to the same subscript identifiers as that for *y*_*ijklm*_. The typical distributional assumptions in mixed models are such that each of the three sets of random effects are NIID with their own VC; that is, *r*(*α* *β*)_*l*:*ij*_ ~ NIID(0, *σ*_*R*(*AB*)_^2^), *s*(*β*)_*m*:*j*_ ~ NIID(0, *σ*_*S*(*B*)_^2^), and *e*_*ijklm*_ ~ NIID(0, *σ*_*E*_^2^). As clearly demonstrated by Dobbin et al. [31] and based on our experiences, dye effects should be modeled in (1), even after using global normalization procedures such as loess [15], as gene-specific dye effects are common. Nevertheless, one would not normally anticipate interaction effects between dye and other treatment factors (e.g., inoculate or time), and hence these effects are not specified in (1).

It should be somewhat apparent from the A-loop design of Figure 2 why the nesting or hierarchical specifications are specified as such for the random effects. For example, although each pool or replicate is labeled twice, once with each dye, each pool is still part of or nested within the same inoculate by time combination such that samples or replicates are specified to be nested within inoculate by time. Similarly, arrays are nested within times since each array is associated with only one particular level of time; that is, different times are never directly compared or connected within arrays. Hence, one should intuitively recognize from Figure 2 that there would be greater precision for inferring upon inoculate effects than for time effects using the A-loop design. That is, the variability due to arrays is completely confounded with time differences such that it partly defines the experimental unit or replicate for time.

### 3.3. Classical ANOVA

The complex nature of different levels of replication in the A-loop this design is further confirmed in the classical analysis of variance or ANOVA [21] for this design in Table 1. However, as demonstrated later, classical ANOVA is not necessarily equivalent to a more optimal GLS or mixed model analysis [32]; in fact, estimates of treatment effects based on classical ANOVA are simply equivalent to OLS estimates where all factors are treated as fixed. Nevertheless, the classical ANOVA table, when extended to include expected mean squares (EMS), is instructive in terms of identifying different levels of replication and hence experimental error.

Classical ANOVA is based on equating sums of squares (SS), also called quadratic forms, to their expectations; typically this involves equating mean squares (MS), being SS divided by their degrees of freedom (*ν*), to their EMS. For completely balanced designs, there is generally one universal manner in which these quadratic forms, and hence the ANOVA table, are constructed [19, 22]. However, for unbalanced designs, such as all of or even just the A-loop component of Figure 2, there are a number of ways of constructing different quadratic forms and hence different ways of constructing ANOVA tables for the same set of data [21, 32]. The most common ANOVA strategy is based on the use of type III quadratic forms as in Table 1 whereby the SS for each factor is adjusted for every other factor in the model. More details on type III and alternative ANOVA quadratic forms for unbalanced data can be found in Milliken and Johnson [21] and Searle [33].

Table 1 conceptually illustrates the basic components of an ANOVA table; again, for every term, say X, in a statistical model like (1), there is a sum of squares (SS_*X*_), degrees of freedom (*v*_*X*_), mean square (MS_*X*_ = SS_*X*_/*v*_*X*_), and expected mean square (EMS_*X*_). Generally, ANOVA tests on fixed effects are of greatest interest; for example, inoculate, time, and inoculate by time interaction. The correct *F* ratio test statistic for any fixed effects term in the ANOVA table is constructed such that its MS and a denominator MS have the same EMS if the null hypothesis is true; that is, that there are truly no effects for that particular term. In statistical parlance, no effects for a term *X*, whether that pertains to the main effects of a factor or the interaction effects between two or more factors, is synonymous with its corresponding *noncentrality parameter* $\left(\varphi_{X}\right)$ being equal to zero; that is, there is no signal due to that model term [32].

Consider, for example, the test for the main effects of inoculate denoted as Factor A in Table 1. If the main effects of inoculate are nonexistent, that is, there are no overall or marginal mean differences between any of the inoculates, then $\varphi_{A}=0$ . It should be clearly noted that when $\varphi_{A}=0$ , the EMS for inoculate matches with the EMS for replicate within inoculate and time, denoted as rep(inoculate*time) in Table 1. In other words, rep(inoculate*time) is said to be the denominator or *error* term for the main effects of inoculate such that rep(inoculate*time) defines the experimental unit or the biological replicate for inoculate effects. Hence, the correct *F* statistic for testing inoculate effects, as demonstrated from Table 1, is *F*_*A*_ = MS_*A*_/MS_*R*(*AB*)_ based on *v*_*A*_ numerator and *v*_*R*(*AB*)_ denominator degrees of freedom. It should also be observed that this same error term or experimental unit would be specified as the denominator MS term for the ANOVA *F*-test on inoculate by time interaction effects, denoted as inoculate*time in Table 1. That is, when the corresponding noncentrality parameter $\varphi_{AB}=0$, both inoculate*time and rep(inoculate*time) share the same EMS such that the correct *F* statistic for testing this interaction is *F*_*AB*_ = MS_*AB*_/MS_*R*(*AB*)_ based on *v*_*AB*_ numerator and *v*_*R*(*AB*)_ denominator degrees of freedom.

It was previously noted from the A-loop design of Figure 2 that inference on the main effects of time (Factor B) should be less precise than that for the main effects of inoculate. In other words, the size of the experimental unit should be larger for time effects since arrays are nested within levels of time whereas levels of inoculate treatments are directly compared within arrays. This is further demonstrated in Table 1 by the EMS for time with $\varphi_{B}=0$ , being larger than that for inoculate effects with $\varphi_{A}=0$ , under the corresponding true null hypotheses of no main effects for either factor. In fact, the experimental error term for time is composite of both rep(inoculate*time) and arrays(time) such that marginal mean comparisons between the three times, 2, 8, and 24 hours, will be affected by more noise than marginal mean comparisons between the three inoculates which were directly and indirectly connected within arrays.

Note that under the null hypothesis of no time effects $(\varphi_{B}=0)$ , there is no one other MS that shares the same EMS *σ*_*E*_^2^ + 2*σ*_*R*(*AB*)_^2^ + 2*σ*_*S*(*B*)_^2^ that would allow one to readily construct an ANOVA *F*-statistic for the main effects of time. Satterthwaite [34] provided a solution to this problem by proposing the “synthesis” of a denominator *MS*, call it MS*, as being a linear combination of *q* random effects MS:

(2) $${\text{MS}}^{*}=a_{1}{\text{MS}}_{1}+a_{2}{\text{MS}}_{2}+a_{3}{\text{MS}}_{3}+\cdots +a_{q}{\text{MS}}_{q},$$

where *a*_1_, *a*_2_,…, *a*_*q*_ are known coefficients such that MS* has the same expectation as that for a certain model term *X* having mean square MS_*x*_ under the null hypothesis $(\varphi_{B}=0)$ . Then *F* = MS_*X*_/MS* is approximately distributed as a random variable from a central *F* distribution with *v*_*X*_ numerator and v* denominator degrees of freedom, where

(3) $$\begin{array}{ll} & v^{*}=\frac{{\left({\text{MS}}^{*}\right)}^{2}}{\theta }, \end{array}$$

with *θ* denoting (*a*_1_MS_1_)^2^/*v*_1_ + (*a*_2_MS_2_)^2^/*v*_2_ + (*a*_3_MS_3_)^2^/*v*_3_ + ⋯ + (*a*_*q*_MS_*q*_)^2^/*v*_*q*_. 

In our example, consider the synthesized MS* = 4/3MS_*R*(*AB*)_ + 4/3MS_*S*(*B*)_− 5/3MSE as being a linear combination of the MS for rep(inoculate*time), array(time), and residual. With reference to (2), MS* is then a linear function of *q* = 3 different MS with *a*_1_ = 4/3, *a*_2_ = 4/3, and *a*_3_ = −5/3. Using the EMS for these three MS provided from Table 1 as (*σ*_*E*_^2^ + 1.5*σ*_*R*(*AB*)_^2^), (*σ*_*E*_^2^ + 1.5*σ*_*S*(*B*)_^2^), and *σ*_*E*_^2^, respectively, it should be readily seen that the expectation of MS* is then

(4) $$\begin{array}{ll} {\text{EMS}}^{*} & =\frac{4}{3}\left(\sigma_{E}^{2}+1.5\sigma_{R(AB)}^{2}\right)+\frac{4}{3}\left(\sigma_{E}^{2}+1.5\sigma_{S(B)}^{2}\right)-\frac{5}{3}\sigma_{E}^{2}\\ & =\sigma_{E}^{2}+2\sigma_{R(AB)}^{2}+2\sigma_{S(B)}^{2}. \end{array}$$

That is, MS* shares the same EMS as that for time in Table 1 when $\varphi_{B}=0$ . Hence, a suitable *F* statistic for inferring upon the main effects of time would be *F*_*B*_ = MS_*B*_/MS*.

**Figure 3:**
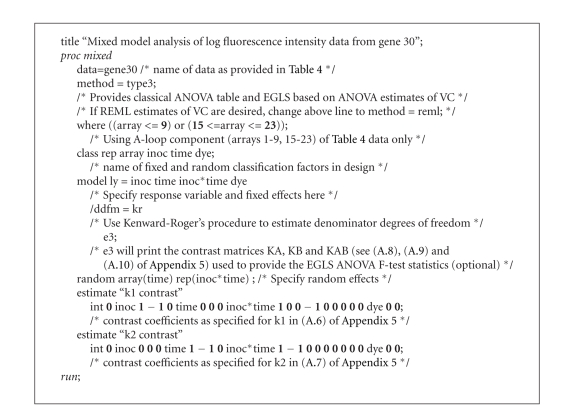
SAS code for classical ANOVA and EGLS inference. Comments describing purpose immediately provided after corresponding code between /* and*/ as with a regular SAS program. EGLS based on REML would simply involve substituting *method = reml* for *method = type3* in the third line of the code.

To help further illustrate these concepts, let us conduct the ANOVA on the data generated from the A-loop design of Figure 2 for ID_REF #30 from Zou et al. [20]; that is, using data from arrays 1–9 and 15–23 as provided in Table 4. The classical ANOVA table using the *method=type3* option of the popular mixed-model software SAS PROC MIXED [35] for that particular gene is provided in Table 2; SAS code for all statistical analysis presented in this paper is provided in Figure 3 and also available for download, along with the data in Table 4, from http://www.msu.edu/~tempelma/ijpg2008.sas. As noted previously, the correct denominator MS term for testing the main effects of inoculate is replicate within inoculate by time. Hence, the corresponding *F* statistic = MS_*A*_/MS_(*R*(*AB*))_ = *F*_*A*_ = 0.356/0.114 = 3.13, with *v*_*A*_ = 2 numerator and *v*_*R*(*AB*)_ = 6 denominator degrees of freedom leading to a *P*-value of 0.1172. Similarly, for the inoculate*time interaction, the appropriate F-test statistic is MS_*AB*_/MS_(*R*(*AB*))_ = *F*_*AB*_ = 0.157/0.114 = 1.38, with *v*_*AB*_= 6 numerator and *v*_*R*(*AB*)_ = 6 denominator degrees of freedom leading to a *P*-value of 0.3435. Even without considering the control of false discovery rates (FDRs) that involve the joint control of type I errors with respect to the remaining 27 683 genes, it seems apparent that neither the main effects of inoculate nor the interaction between inoculate and time would be statistically significant for gene ID_REF #30.

The synthesized denominator MS* for time effects is MS* = 4/3MS_*R*(*AB*)_ + 4/3MS_*S*(*B*)_ − 5/3MSE = 4/3(0.114) + 4/3(0.361) − 5/3(0.034) = 0.576. The estimated degrees of freedom for this synthesized MS using (3) is then

(5) $$\begin{array}{ll} v^{*} & =\frac{{\left({\text{MS}}^{*}\right)}^{2}}{{\left(a_{1}{\text{MS}}_{R\left(AB\right)}\right)}^{2}/v_{R\left(AB\right)}+{\left(a_{2}{\text{MS}}_{S\left(B\right)}\right)}^{2}/v_{S\left(B\right)}+{\left(a_{3}{\text{MS}}_{E}\right)}^{2}/v_{E}}\\ & =\frac{{\left(0.576\right)}^{2}}{{\left(\left(4/3\right)\cdot 0.114\right)}^{2}/6+{\left((4/3)\cdot 0.361\right)}^{2}/12+{\left(-(5/3)\cdot 0.034\right)}^{2}/5}\\ & =13.97. \end{array}$$

Hence, the main effects of time, appropriate *F*-test statistic is MS_*B*_/MS* = *F*_*B*_ = 1.88/0.576 = 3.27, with *v*_*B*_ = 2 numerator and $\begin{array}{c} v^{*} \end{array}=13.97$ denominator degrees of freedom leading to a *P*-value of 0.0683 as also reported in the SAS output provided in Table 2.

### 3.4. Mixed model analysis

Although the classical ANOVA table is indeed instructive in terms of illustrating the different levels of variability and experimental error, it is not the optimal statistical analysis method for a mixed effects model, especially when the design is unbalanced. A mixed-model or GLS analysis more efficiently uses information on the design structure (i.e., random effects) for inferring upon the fixed treatment structure effects [27, 32].

Unfortunately, GLS, in spite of its optimality properties, is generally not attainable with real data because the VC (e.g., *σ*_*R*(*AB*)_^2^, *σ*_*S*(*B*)_^2^, and *σ*_*E*_^2^) must be known. Hence, the VC must generally be estimated from the data at hand. There are a number of different methods that are available for estimating VC in mixed models [22]. The classical ANOVA method is based on equating MS to their EMS in the ANOVA table. For example, using the bottom row of Table 1, the EMS of MSE is *σ*_*E*_^2^. So then using the numerical results for ID_REF #30 from Table 2, the type III ANOVA estimate of *σ*_*E*_^2^ is simply σ^E2=MSE=0.034. Now work up one row further in Table 1 to the term array(time). Equating MS_*S*(*B*)_ = 0.361 from the same corresponding row in Table 2 to its EMS of *σ*_*E*_^2^ + 1.5*σ*_*S*(*B*)_^2^ using ${\hat{\sigma }}_{E}^{2}=0.034$ gives ${\hat{\sigma }}_{S\left(B\right)}^{2}=0.218$. Finally, work up one more (i.e., third to last) row in both tables. Equating MS_*R*(*AB*)_ = 0.114 from Table 2 to its EMS of *σ*_*E*_^2^ + 1.5*σ*_*R*(*AB*)_^2^ using ${\hat{\sigma }}_{E}^{2}=0.034$ leads to ${\hat{\sigma }}_{R\left(AB\right)}^{2}=0.053$. So array variability *σ*_*S*(*B*)_^2^ is estimated to be roughly four times larger than the biological variability *σ*_*R*(*AB*)_^2^ which, in turn, is estimated to be somewhat larger than residual variability *σ*_*E*_^2^ for ID_REF #30.

Recall that with unbalanced designs, quadratic forms are not unique such that ANOVA estimators of VC will not be unique either. Nevertheless, type III quadratic forms are most commonly chosen as then the SS for each term is adjusted for all other terms, as previously noted. Although ANOVA estimates of VC are unbiased, they are not efficient nor optimal in terms of estimates having minimum standard error [25]. Restricted maximum likelihood (REML) is a generally more preferred method of VC estimation [22, 36, 37] and is believed to have more desirable properties. Nevertheless, the corresponding REML estimates ${\hat{\sigma }}_{E}^{2}=0.033$, ${\hat{\sigma }}_{S\left(B\right)}^{2}=0.258$ and ${\hat{\sigma }}_{R\left(AB\right)}^{2}=0.061$ for ID_REF #30 are in some qualitative agreement with the previously provided ANOVA estimates.

Once the VCs are estimated, they are substituted for the true unknown VCs to provide the “estimated” GLS or EGLS of the fixed effects. It is important to note that typically EGLS = GLS for balanced designs, such that knowledge of VC is somewhat irrelevant for point estimation of treatment effects. However, the same is generally not true for unbalanced designs, such as either the A-loop design derived from Figure 2 or even the interwoven loop design from Figure 1. Hence, different methods of VC estimation could lead to different EGLS estimates of treatment effects as we demonstrate later. Suppose that it was of interest to compare the various mean responses of various inoculate by time group combinations in the duplicated A-loop design example. Based on the effects defined in the statistical model for this design in (1), the true mean response for the *i*th inoculate at the *j*th time averaged across the two dye effects (*δ*_1_ and *δ*_2_) can be written as

(6) $$\mu_{ij_{\cdot }}=\mu +\alpha_{i}+\beta_{j}+\alpha \beta_{ij}+0.5\delta_{1}+0.5\delta_{2}.$$

If the levels are, say, ordered alphanumerically, the mean difference between inoculate *i* = 1(*M*) and *i* = 2(*R*) at time *j* = 1 (2 hours) is specified as *μ*_11._ − *μ*_21._. Using (6), this difference written as a function of the model effects is then *μ*_11._ − *μ*_21._ = (*μ* + *α*_1_ + *β*_1_ + *α* *β*_11_ + 0.5*δ*_1_ + 0.5*δ*_2_) − (*μ* + *α*_2_ + *β*_1_ + *α* *β*_21_ + 0.5*δ*_1_ + 0.5*δ*_2_) = *α*_1_ − *α*_2_ + *α* *β*_11_ − *α* *β*_21_. Similarly, the mean difference *μ*_11._ − *μ*_12._ between time *j* = 1 (2 hours) and time *j* = 2 (8 hours) for inoculate *i* = 1(*M*) could be derived as *β*_1_ − *β*_2_ + *α* *β*_11_ − *α* *β*_12_. Note that these two comparisons or *contrasts* can be more elegantly written using matrix algebra notation. A better understanding of contrasts is useful to help determine the correct standard errors and statistics used to test these contrasts, including how to write the corresponding SAS code. Hence, a matrix algebra approach to hypothesis testing on contrasts is provided in Appendix 5 that complements the SAS code provided in Figure 3. For now, however, we simply use the “hat” notation (  ^ ) in referring to the EGLS estimates of these two contrasts as ${\hat{\mu }}_{11.}-{\hat{\mu }}_{21.}$ and ${\hat{\mu }}_{11.}-{\hat{\mu }}_{12.},$ respectively.

As we already intuitively noted from the A-loop design of Figure 2, inference on *μ*_11._ − *μ*_21._ should be much more precise than that for *μ*_11._ − *μ*_12._ since inoculates are compared within arrays whereas times are not. This distinction should then be reflected in a larger standard error for ${\hat{\mu }}_{11.}-{\hat{\mu }}_{12.}$ than for ${\hat{\mu }}_{11.}-{\hat{\mu }}_{21.}$. Indeed, using the REML estimates of VC for EGLS inference, this is demonstrated by se^ (μ^11.−μ^21.)=0.2871 whereas se^ (μ^11.−μ^12.)=0.4085 for ID_REF #30. However, these standard errors are actually slightly understated since they do not take into account the uncertainty of the VC estimates as discussed by Kackar and Harville [38]. Kenward and Roger [39] derive a procedure to take this uncertainty into account which is part of the SAS PROC MIXED implementation using the option *ddfm=kr* [35] as indicated in Figure 3. Invoking this option raises the two standard errors accordingly, albeit very slightly, to se^ (μ^11.−μ^21.)=0.2878 and se^ (μ^11.−μ^12.)=0.4088.

Now, the denominator degrees of freedom for inference on these two contrasts should also differ given that the nature of experimental error variability somewhat differs for inoculate comparisons as opposed to time comparisons as noted previously from Figure 2. However, with EGLS, there are no SS and hence no corresponding MS or EMS expression for each main effects or interaction term in the model, such that determining the correct test statistic and degrees of freedom is somewhat less obvious than with the previously described classical ANOVA approach [32]. Giesbrecht and Burns [40] introduced a procedure for estimating the denominator degrees of freedom for EGLS inference which, again, is invoked with the *ddfm=kr* option of SAS PROC MIXED. Using this option along with REML estimation of VC for the analysis of ID_REF #30, the estimated degrees of freedom for ${\hat{\mu }}_{11.}-{\hat{\mu }}_{21.}$ is 5.28 whereas that for ${\hat{\mu }}_{11.}-{\hat{\mu }}_{12.}$ is 17.0.

Contrasts are also used in EGLS to provide ANOVA-like *F* tests for the overall importance of various fixed effects; more details based on the specification of contrast matrices to test these effects are provided in Appendix 5. For example, denote the marginal or overall mean of inoculate *i* averaged across the 3 times and 2 dyes as *μ*_*i*.._ = (1/3)∑_*j*=1_^3^ *μ*_*ij*._. The *v*_*A*_ = 2 numerator degrees of freedom hypothesis test for the main effects of inoculates can be written as a combination of two complementary contrasts (A1) H_0_ : *μ*_1.._ − *μ*_3.._ = 0 and (A2) H_0_ : *μ*_2.._ − *μ*_3.._ = 0; that is, if both contrasts are 0, then obviously H_0_ : *μ*_2.._ − *μ*_3.._ = 0 is also true such that then H_0_:*μ*_1.._ = *μ*_2.._ = *μ*_3.._ is true. Similarly, let us suppose that one wished to test the main effects of times (Factor B). Then, it could be readily demonstrated that the corresponding hypothesis test can also be written as a combination of *v*_*B*_ = 2 complementary contrasts: (B1) H_0_ : *μ*_.1._ − *μ*_.3._ = 0 and (B2) H_0_ : *μ*_.2._ − *μ*_.3._ = 0, where *μ*_.*j*._ = (1/3)∑_*i*=1_^3^ *μ*_*ij*._ denotes the marginal mean for the *j*th level of Factor B; that is, the *j*th time. If both component hypotheses (B1) and (B2) are true, then H_0_:*μ*_.1._ = *μ*_.2._ = *μ*_.3._ = 0 is also true thereby defining the composite *v*_*B*_ = 2 numerator degrees of freedom hypothesis test for the main effects of Factor B.

Now the interaction between inoculate and time is a *v*_*AB*_ = *v*_*A*_ *v*_*B*_ = 2*2 = 4 numerator degrees of freedom test as previously noted from Tables 1 and 2, suggesting that there are 4 complementary contrasts that jointly test for the interaction of the two factors. Of course, it is also well known that the interaction degrees of freedom is typically the product of the main effects degrees of freedom for the two factors considered. Two of the four degrees of freedom for the interaction involve testing whether or not the mean difference between inoculates 1 and 3 is the same within time 1 as it is within time 3, that is, (AB1) H_0_ : *μ*_11._ − *μ*_31._ − (*μ*_13._ − *μ*_33._) = 0, and whether or not the mean difference between inoculates 2 and 3 is the same within time 1 as it is within time 3; that is, (AB2) H_0_ : *μ*_21._ − *μ*_31._ − (*μ*_23._ − *μ*_33._) = 0. If both hypotheses (AB1) and (AB2) are true then it should be apparent that H_0_ : *μ*_11._ − *μ*_21._ − (*μ*_13._ − *μ*_23._) = 0 is also true; that is, the mean difference between inoculates 1 and 2 is the same within time 1 as it is within time 3. The remaining two degrees of freedom for the interaction involve testing whether or not the mean difference between inoculates 1 and 3 is the same within time 2 as it is within time 3; that is, (AB3) H_0_ : *μ*_12._ − *μ*_32._ − (*μ*_13._ − *μ*_33._) = 0, and whether or not the mean difference between inoculates 2 and 3 is the same within time 2 as it is within time 3; that is, (AB4) H_0_ : *μ*_22._ − *μ*_32._ − (*μ*_23._ − *μ*_33._) = 0. If both hypotheses (AB3) and (AB4) are true then H_0_ : *μ*_12._ − *μ*_22._ − (*μ*_13._ − *μ*_23._) = 0 is also true. Hence, contrasts AB1, AB2, AB3, and AB4 completely define the four components or numerator degrees of freedom for the interaction between Factors A and B. That is, the test for determining whether or not the mean differences between all levels of A are the same within each level of B, and vice versa, can be fully characterized by these four complementary contrasts.

The EGLS statistics used for testing the overall importance of these main effects or interactions are approximately distributed as *F*-random variables with the numerator degrees of freedom defined by the number of complementary components or contrasts as previously described; refer to Appendix 5 and elsewhere [27, 32, 35] for more details. Now, the denominator degrees of freedom for each contrast are dependent upon the design and can be determined based on that using classical ANOVA as in Table 1 or by a multivariate extension of the Satterthwaite-based procedure proposed by Fai and Cornelius [41]; again this option is available as *ddfm=kr* using SAS PROC MIXED (Figure 3).

Unfortunately, much available software used for mixed model analysis of microarray data does not carefully take into consideration that various fixed effects terms of interest may have different denominator degrees of freedom when constructing *F* test statistics. In fact, a typical strategy of such software is to assume that *v*_*E*_ (i.e., the residual degrees of freedom) is the denominator degrees of freedom for all tests. This strategy is denoted as the “residual” method for determining denominator degrees of freedom by Spilke et al. [36] who demonstrated using simulation work that the use of the residual method can substantially inflate type I error rate for EGLS inference on fixed effects; in other words, the number of false-positive results or genes incorrectly declared to be differentially expressed between treatments would be unduly increased. Spilke et al. [36] further demonstrated that use of the Kenward-Rogers’ method for degrees of freedom estimation and control for uncertainty on VC provided best control of the nominal confidence interval coverage and type I error probabilities.

### 3.5. Impact of method of variance component estimation on EGLS

It was previously noted that the estimated standard errors for EGLS on two contrasts *μ*_11._ − *μ*_21._ and *μ*_11._ − *μ*_12._ were se^ (μ^11.−μ^21.)=0.2878 and se^ (μ^11.−μ^12.)=0.4088, respectively, when REML was used to estimate the variance components for ID_REF #30. If the VC estimates are computed using type III ANOVA, then these estimated standard errors would differ accordingly; that is, se^ (μ^11.−μ^21.)=0.2752 and se^ (μ^11.−μ^12.)=0.3828, respectively. What perhaps is even more disconcerting is that the estimates of *μ*_11._ − *μ*_21._ and *μ*_11._−*μ*_12._ also differ between the two EGLS inferences; for example, using REML, ${\hat{\mu }}_{11.}-{\hat{\mu }}_{21.}=0.1328$ and ${\hat{\mu }}_{11.}-{\hat{\mu }}_{12.}=-0.0881$ whereas using ANOVA ${\hat{\mu }}_{11.}-{\hat{\mu }}_{21.}=0.1298$ and ${\hat{\mu }}_{11.}-{\hat{\mu }}_{12.}=-0.0873$.

The overall EGLS tests for ID_REF #30 for testing the main effects of inoculate, time and their interaction as based on the previously characterized complementary contrasts are provided separately for ANOVA versus REML estimates of VC in Table 3; this output is generated as type III tests using the SAS code provided in Figure 3. From here, it should be clearly noted that conclusions upon the overall importance of various fixed effects terms in (1) as derived from EGLS inference subtly depend upon the method of VC estimation; for example, the EGLS *P*-values in Table 3 tend to be several points smaller using ANOVA compared to REML; furthermore, note the differences in the estimated denominator degrees of freedom between the two sets. Naturally, this begs the question as to which method of VC estimation should be used?

In completely balanced designs, ANOVA and REML lead to identical estimates of VC and identical EGLS inference, provided that all ANOVA estimates of VC are positive. ANOVA estimates of VC that are negative are generally constrained by REML to be zero, thereby causing a “ripple” effect on REML estimates of other VC and subsequently on EGLS inference [42]. As noted previously, REML does tend to outperform most other methods for various properties of VC estimation [37]. Furthermore, there is evidence that EGLS based on ANOVA leads to poorer control of type I error rate for inference on fixed effects compared to EGLS based on REML in unbalanced data structures [36]. However, Stroup and Littell [42] concluded that EGLS using REML may sometimes lead to inference on fixed effects that is too conservative (i.e., actual error rates less than nominal type I error rate) again due to the nonnegative REML restrictions on the VC estimates and associated ripple effects. This issue warrants further study given that it has implications for control of FDR which are most commonly used to control the rate of type I errors in microarray studies [43]. Estimation of FDR inherently depends upon the distribution of *P*-values for treatment effects across all genes such that even mild perturbations on this distribution have potential bias implications for control of false-positive rates.

## 4. OTHER ISSUES FOR THE DESIGN ANALYSIS INTERFACE

### 4.1. Log ratio versus log intensity modeling

Recent work on the optimization and comparison of various efficient microarray designs have been based on the assumption of OLS inference; that is, no random sources of variability other than residuals are considered [2, 8, 9, 13]. While this observation may seem to be counterintuitive given that the arguments laid out in this review for the need of (E)GLS to analyze efficient designs, it is important to note at least a couple of things. First, virtually all of the work on design optimization has been based on the assumption that a sample or pool is used only once; the corresponding interwoven loop designs in such cases [13] have been referred to as classical loop designs [10, 19]. However, sometimes two or more aliquots from each sample are used in separate hybridizations [20, 23] such as the A-loop design, example used in this review; the corresponding designs are connected loop designs [10, 19] that require the specification of random biological replicate effects separate from residual effects as previously noted.

Secondly, almost all of the design optimization work has been based on the use of Cy3/Cy5 log ratios as the response variables rather than dye-specific log intensities as used in this review. This data reduction, that is, from two fluorescence intensities to one ratio per spot on an array, certainly eliminates array as a factor to specify in a linear model. However, the use of log ratios can severely limit estimability and inference efficiency of certain comparisons. Suppose that instead of using the 36 log intensities from the duplicated A-loop design from arrays 1–9 and 15–23 of Table 4, we used the derivative 18 Cy3/Cy5 log ratios as the response variables. For example, the two corresponding log_2_Cy3 and Cy5 fluorescence intensities for array 1 from Table 4 are 13.9946 and 14.3312. The Cy3/Cy5 log ratio is then the difference or −0.3316 corresponding to a fold change of 2^−0.3316^ = 0.795. Using log ratios as their response variables, Landgrebe et al. [9] concluded that it was impossible to infer upon the main effects of Factor B (e.g., time) in the A-loop design. However, as we demonstrated earlier, it is possible to infer upon these effects using ANOVA or EGLS analysis on the log intensities. Jin et al. [18] similarly illustrate the utility of log intensity analysis in a split plot design that would not otherwise have been possible using log ratios. Milliken et al. [14] provide much more extensive mixed modeling details on the utility of log intensity analysis in nested or split-plot microarray designs similar to the A-loop design.

The relative efficiency of some designs may be seen to depend upon the relative magnitude of biological to technical variation [10, 44]; sometimes it is only possible to separately estimate these two sources of variability using log intensities rather than log ratios thereby requiring the use of (E)GLS rather than OLS. In fact, analysis of log intensities using mixed effects model appears to be not only more flexible than log-ratio modeling but is statistically more efficient in recovering more data information [1, 45]. That is, as also noted by Milliken et al. [14], treatment effects are more efficiently estimated by combining intraarray and interarray information in a mixed model analysis when an incomplete block design is used, and arrays are explicitly included as random effects by analyzing log intensities rather than log ratios.

### 4.2. Choosing between efficient experimental designs using mixed models

There are a number of different criteria that might be used to choose between different designs for two-color microarrays. We have already noted that the interwoven loop design in Figure 1 is A-optimal for pairwise comparisons between 9 treatment groups. A-optimality has been criticized for microarray studies because it chooses designs with improved efficiency for certain contrasts at the expense of other perhaps more relevant contrasts and further depends upon the parameterization of the linear model [1, 6, 9]; other commonly considered types of optimality criteria are possible and further discussed by Wit et al. [13] and Landgrebe et al. [9]. At any rate, it is somewhat possible to modify A-optimality to explicitly take into account a particular set of scientific questions [13]; furthermore, optimization with respect to one criterion will generally be nearly optimal for others.

For one particular type of optimality criterion, Landgrebe et al. [9] demonstrated that the A-loop design has the best relative efficiency compared to other designs for inference on the main effects of Factor A and the interaction effects between A and B although the main effects of Factor B could not be inferred upon using an analysis of log ratios as previously noted. How does the A-loop design of Figure 2 generally compare to the interwoven loop design of Figure 1 if a 3 × 3 factorial treatment structure is imposed on the 9 treatments as implied by the same labels as used in Figure 2? Suppose that Figure 1 is a connected interwoven loop design [10] in the sense that the outer loop of Figure 1 (dashed arrows) connects one biological replicate for each of 9 groups whereas the inner loop of Figure 1 (solid arrows) connects a second biological replicate for each of the 9 groups. Then this design would consume 18 biological replicates and 18 arrays, thereby providing a fair comparison with the duplicated A-loop design of Figure 2.

Recall that Figure 1 is A-optimized for pairwise comparisons between all 9 groups. It is not quite clear what implications this might have for statistical efficiency for the constituent main effects of *A*(*v*_*A*_ = 2), *B*(*v*_*B*_ = 2), and the effects of their interaction A*B(*v*_*AB*_ = 4); note, incidentally, that these degrees of freedom independently sum to 8 as required for 9 groups. As duly noted by Altman and Hua [1], pairwise comparisons between all 9 groups may be not as important as various main effects or interaction contrasts with a factorial treatment structure arrangement. Although, as noted earlier, Figure 1 is symmetric with respect to the treatment labels, the classical ANOVA table for this interwoven loop design would be even more complicated (not shown) than that presented for the A-loop design since there is not one single denominator MS that would serve as the experimental error term for inoculate, time or inoculate by time effects!

One should perhaps compare two alternative experimental designs having the same factorial treatment structure, but a different design structure, for contrasts of highest priority, choosing those designs where such contrasts have the smaller standard error. Let us consider the following comparisons: *μ*_1.._ − *μ*_3.._, *μ*_.1._ − *μ*_.3._, and *μ*_11._ − *μ*_31._ − (*μ*_13._ − *μ*_33._); that is, respectively, the overall mean difference between inoculates 1 and 3, the overall mean difference between times 1 and 3, and the interaction component pertaining to the difference between inoculates 1 and 3 within time 1 versus that same difference within time 3. Recall that these contrasts were components of the EGLS tests on the two sets of main effects and the interaction and previously labeled as (A1), (B1), and (AB1), respectively.

Now the comparison of efficient designs for the relative precision of various contrasts will generally depend upon the relative magnitude of the random effects VC as noted recently by Hedayat et al. [44] and for various microarray design comparisons [10]. Suppose the “true” variance components for *σ*_*E*_^2^, *σ*_*R*(*AB*)_^2^, and *σ*_*S*(*B*)_^2^ were 0.03, 0.06, and 0.25, comparable to either set of estimates provided previously on ID_REF #30 from Zou et al. [20]. The linear mixed model for analyzing data generated from Figure 1 would be identical to that in (1) except that arrays would no longer be specified as being nested within times. For the interwoven loop design of Figure 1, the standard errors for each of the three contrasts are $se\left({\hat{\mu }}_{1..}-{\hat{\mu }}_{3..}\right)\;=\;0.18$, $se\left({\hat{\mu }}_{.1.}-{\hat{\mu }}_{.3.}\right)\;=\;0.21$, and $se\left({\hat{\mu }}_{11.}-{\hat{\mu }}_{31.}-\left({\hat{\mu }}_{13.}-{\hat{\mu }}_{33.}\right)\right)\;=\;0.43$ whereas for the A-loop subdesign of Figure 2, the corresponding standard errors are $se\left({\hat{\mu }}_{1..}-{\hat{\mu }}_{3..}\right)\;=\;0.16$, $se\left({\hat{\mu }}_{.1.}-{\hat{\mu }}_{.3.}\right)\;=\;0.33$, and $se\left({\hat{\mu }}_{11.}-{\hat{\mu }}_{31.}-\left({\hat{\mu }}_{13.}-{\hat{\mu }}_{33.}\right)\right)\;=\;0.40$. So whereas the optimized design in Figure 1 using Wit et al. [13] provided a substantial improvement for the estimation of overall mean time differences, the A-loop design is indeed more efficient for inferring upon the main effects of inoculate and the interaction between inoculate and time. Hence, the choice between the two designs would reflect a matter of priority for inference on the various main effects and their interactions. It should be carefully noted as demonstrated by Tempelman [10], that designs leading to lower standard errors for certain comparisons do not necessarily translate to greater statistical power as the denominator degrees of freedom for various tests may be substantially different between the two designs.

### 4.3. Unbalanced designs and shrinkage estimation

Shrinkage or empirical Bayes (EB) estimation is known to improve statistical power for inference on differential gene expression between treatments in microarray experiments [46]. Shrinkage-based estimation is based on the well-established hierarchical modeling concept that more reliable inferences on gene-specific treatment differences are to be attained by borrowing information across all genes [47, 48]. Typically, such strategies have involved improving estimation of standard errors of gene-specific treatment differences by “shrinking” gene-specific variances towards an overall mean or other measure of central tendency. However, most shrinkage estimation procedures have been developed for fixed effects models, that is, for simple experimental designs having a treatment structure but no or very limited design structure, or even treating all design structure factors as fixed [30]. Currently popular shrinkage estimation procedures [49–51] are certainly appropriate for many designs based on one-color Affymetrix systems or for common reference designs. Other proposed shrinkage procedures have facilitated extensions to very special cases of nested designs [47], including some based on rather strong modeling assumptions such as a constant correlation of within-array replicate spots across all genes [52] or a design structure facilitating the use of permutation testing [29]. However, virtually none of the procedures proposed thus far are well adapted to handle unbalanced designs such as the A-loop design where different sizes of experimental units need to be specified for different treatment factors; hence investigators should proceed with caution when using shrinkage estimation for unbalanced mixed-model designs.

## 5. CONCLUSIONS

We have provided an overview of the use of mixed linear model analysis for the processing of unbalanced microarray designs, given that most efficient incomplete block designs for microarrays are unbalanced with respect to various comparisons. We strongly believe that much mixed-model software currently available for the analysis of microarrays does not adequately address the proper determination of error terms and/or denominator degrees of freedom for various tests. This would be particularly true if we had chosen to analyze all of the data for ID_REF #30 in Table 4 from Zou et al. [20] based on all of the 2 × 14 hybridizations depicted in Figure 2. Even then, the size of the standard errors and estimated degrees of freedom would still be seen to be somewhat different for estimating the main effects of times compared to estimating the main effects of inoculates given the lower degree of within-array connectivity between the various levels of time as illustrated in Figure 2. If inferences on various comparisons of interest are not conducted correctly in defining a list of differently expressed genes, all subsequent microarray analysis (e.g., FDR estimates, gene clustering, gene class analysis, etc.) are absolutely futile.

We believe that it is useful to choose proven mixed-model software (e.g., SAS) to properly conduct these tests and, if necessary, to work with an experienced statistician in order to do so. We have concentrated our attention on the analysis of a particular gene. It is, nevertheless, straightforward to use SAS to serially conduct mixed-model analysis for all genes on a microarray [53]; furthermore, SAS JMP GENOMICS (http://www.jmp.com/software/genomics/) provides an even more powerful user interface to the mixed model analysis of microarray data.

## Figures and Tables

**Table 1:** Classical ANOVA of log intensities for duplicated A-loop design component of Figure 2 for any particular gene using (1).

Source	SS*	*d* *f* ^†^	Mean square	Expected mean square
Inoculate	SS_*A*_	*v*_*A*_	MS_*A*_ = SS_*A*_/*v*_*A*_	*σ*_*E*_^2^ + 1.5*σ*_*R*(*A*·*B*)_^2^ + ${\varphi_{A}}^{‡}$
Time	SS_*B*_	*v*_*B*_	MS_*B*_ = SS_*B*_/*v*_*B*_	*σ*_*E*_^2^ + 2*σ*_*R*(*A*·*B*)_^2^ + 2*σ*_*S*(*B*)_^2^ + $\varphi_{B}$
Inoculate*time	SS_*AB*_	*v*_*AB*_	MS_*AB*_ = SS_*AB*_/*v*_*AB*_	*σ*_*E*_^2^ + 1.5*σ*_*R*(*A*·*B*)_^2^ + $\varphi_{AB}$
Dye	SS_*D*_	*v*_*D*_	MS_*D*_ = SS_*D*_/*v*_*D*_	*σ*_*E*_^2^ + $\varphi_{D}$
Rep(inoculate*time)	SS_*R*(*AB*)_	*v*_*R*(*AB*)_	MS_*R*(*AB*)_ = SS_*R*(*AB*)_/*v*_*R*(*AB*)_	*σ*_*E*_^2^ + 1.5*σ*_*R*(*A*·*B*)_^2^
Array(time)	SS_*S*(*B*)_	*v*_*S*(*B*)_	MS_*S*(*B*)_ = SS_*S*(*B*)_/*v*_*S*(*B*)_	*σ*_*E*_^2^ + 1.5*σ*_*S*(*B*)_^2^
Error	SS_*E*_	*v*_*E*_	MS_*E*_ = SS_*E*_/*v*_*E*_	*σ*_*E*_^2^

*Sums of squares. ^†^Degrees of freedom. ^‡^ *φ*_*X*_ is the noncentrality parameter for factor *X*. For example, when $\varphi_{A}=0$ , there are no overall mean inoculate differences such that inoculate and Rep(inoculate*time) have the same expected mean square and *F*_*A*_ = MS_*A*_/MS_*R*(*AB*)_ is a random draw from an *F* distribution with *v*_*A*_ numerator and *v*_*R*(*AB*)_ denominator degrees of freedom.

**Table 2:** Classical ANOVA of log intensities for duplicated A-loop design component of Figure 2 on ID_REF #30 from Zou et al. (2005) using output from SAS PROC MIXED (code in Figure 3).

Type 3 analysis of variance							
Source	DF^†^	Sum of squares	Mean square	Expected mean square	Error term	Error DF	*F* value	Pr > F^‡^
Trt	2	0.7123	0.3561	Var(Residual) + 1.5 Var(sample(inoc*time)) + Q(inoc,inoc*time)	MS(sample(inoc*time))	6	3.13	0.1172
Time	2	3.7737	1.8868	Var(Residual) + 2 Var(sample(inoc*time)) + 2Var(array(time)) + Q(time,inoc*time)	1.3333 MS(array(time)) + 1.3333 MS(sample(inoc*time)) − 1.6667 MS(Residual)	13.969	3.27	0.0683
Inoc*time	4	0.6294	0.1573	Var(Residual) + 1.5 Var(sample(inoc*time)) + Q(inoc*time)	MS(sample(inoc*time))	6	1.38	0.3435
Dye	1	0.0744	0.0744	Var(Residual) + Q(dye)	MS(Residual)	5	2.19	0.1989
Rep(inoc*time)	6	0.6826	0.1137	Var(Residual) + 1.5 Var(sample(inoc*time))	MS(Residual)	5	3.35	0.1030
Array(time)	12	4.3330	0.3610	Var(Residual) + 1.5 Var(array(time))	MS(Residual)	5	10.63	0.0085
Residual	5	0.1699	0.0339	Var(Residual)	.	.	.	.

^†^Degrees of freedom. ^‡^ *P*-value.

**Table 3:** EGLS inference on overall importance of fixed effects for ID_REF #30 based on REML versus ANOVA (type III quadratic forms) for estimation of variance components using output from SAS PROC MIXED (code in Figure 3).

		Type 3 tests of fixed effects using REML			Type 3 tests of fixed effects using ANOVA		
Effect	Num DF*	Den DF*	F value	Pr > F^†^	Den DF*	F value	Pr > F^†^
Inoc	2	5.28	3.12	0.1273	6.36	3.48	0.0954
Time	2	17.8	2.81	0.0870	22.8	3.27	0.0563
Inoc*time	4	5.28	1.26	0.3893	6.36	1.38	0.3392
Dye	1	5.43	2.27	0.1879	5.15	2.19	0.1973

*Num Df = numerator degrees of freedom; Den DF = denominator degrees of freedom. ^†^ *P*-value.

**Table 4:** Dataset for ID_REF #30 for all hybridizations (14 arrays/loop x2 loops) in Figure 1 for each of two replicates per 10 inoculate by time groups, fluorescence intensities provided as y, log(base 2) intensities provided as ly.

Obs	array	inoculate	time	rep	dye	y	ly
1	1	R	2	1R2	Cy3	16322.67	13.9946
2	1	M	2	1M2	Cy5	20612.48	14.3312
3	2	M	2	1M2	Cy3	10552.21	13.3653
4	2	S	2	1S2	Cy5	10640.89	13.3773
5	3	S	2	1S2	Cy3	24852.98	14.6011
6	3	R	2	1R2	Cy5	21975.92	14.4236
7	4	R	8	1R8	Cy3	30961.96	14.9182
8	4	M	8	1M8	Cy5	13405.08	13.7105
9	5	M	8	1M8	Cy3	13103.51	13.6777
10	5	S	8	1S8	Cy5	15659.44	13.9347
11	6	S	8	1S8	Cy3	20424.47	14.3180
12	6	R	8	1R8	Cy5	34244.92	15.0636
13	7	R	24	1R24	Cy3	15824.29	13.9499
14	7	M	24	1M24	Cy5	13014.05	13.6678
15	8	M	24	1M24	Cy3	17503.11	14.0953
16	8	S	24	1S24	Cy5	27418.99	14.7429
17	9	S	24	1S24	Cy3	37689.16	15.2019
18	9	R	24	1R24	Cy5	55821.64	15.7685
19	10	S	2	1S2	Cy3	28963.28	14.8219
20	10	S	8	1S8	Cy5	38659.44	15.2385
21	11	S	8	1S8	Cy3	41608.78	15.3446
22	11	S	24	1S24	Cy5	41844.79	15.3528
23	12	R	2	1R2	Cy3	12132.41	13.5666
24	12	R	8	1R8	Cy5	19131.53	14.2237
25	13	R	8	1R8	Cy3	31067.04	14.9231
26	13	R	24	1R24	Cy5	26197.03	14.6771
27	14	N	2	1N2	Cy3	18540.91	14.1784
28	14	M	2	1M2	Cy5	24971.88	14.6080
29	15	R	2	2R2	Cy3	9612.25	13.2307
30	15	M	2	2M2	Cy5	9212.11	13.1693
31	16	M	2	2M2	Cy3	10322.23	13.3335
32	16	S	2	2S2	Cy5	10979.19	13.4225
33	17	S	2	2S2	Cy3	8061.40	12.9768
34	17	R	2	2R2	Cy5	6737.37	12.7180
35	18	R	8	2R8	Cy3	8807.09	13.1044
36	18	M	8	2M8	Cy5	8696.95	13.0863
37	19	M	8	2M8	Cy3	15186.20	13.8905
38	19	S	8	2S8	Cy5	23477.49	14.5190
39	20	S	8	2S8	Cy3	19424.30	14.2456
40	20	R	8	2R8	Cy5	18198.99	14.1516
41	21	R	24	2R24	Cy3	19630.00	14.2608
42	21	M	24	2M24	Cy5	15629.14	13.9320
43	22	M	24	2M24	Cy3	10875.49	13.4088
44	22	S	24	2S24	Cy5	20816.21	14.3454
45	23	S	24	2S24	Cy3	24647.70	14.5892
46	23	R	24	2R24	Cy5	22148.96	14.4350
47	24	S	2	2S2	Cy3	17795.09	14.1192
48	24	S	8	2S8	Cy5	34569.11	15.0772
49	25	S	8	2S8	Cy3	44175.28	15.4310
50	25	S	24	2S24	Cy5	38020.46	15.2145
51	26	R	2	2R2	Cy3	34689.07	15.0822
52	26	R	8	2R8	Cy5	62219.10	15.9251
53	27	R	8	2R8	Cy3	22724.21	14.4719
54	27	R	24	2R24	Cy5	19594.71	14.2582
55	28	N	2	2N2	Cy3	11755.32	13.5210
56	28	M	2	2M2	Cy5	12599.55	13.6211

## APPENDIX

### MATRIX REPRESENTATION OF THE MIXED MODEL ANALYSIS OF THE A-LOOP DESIGN OF ZOU ET AL.

Any mixed model, including that specified in (1), can be written in matrix algebra form:

(A.1) y=Xβ + Zu + e.

Here ****y** = {*y*_*i**j**k**lm*_}** is the vector of all data, ***β*** is the vector of all fixed effects (e.g., inoculate, time, dye, and inoculate by time interaction effects), **u** is the vector of all random effects (e.g., arrays and sample within inoculate by time effects), and **e** = {*e*_*i**j**k**lm*_} is the vector of random residual effects. Furthermore, **X** and **Z** are corresponding incidence matrices that specify the treatment and design structure of the experiment, thereby linking the treatment and design effects, ***β*** and **u**, respectively, to **y**. Note that **y** has a dimension of 36 × 1 for the duplicated A-loop design of Zou et al. [20]. Now ***β*** and **u** can be further partitioned into the effects as specified in (1); for our example,

(A.2) $$\begin{array}{cc} & \beta =[\begin{array}{cccccccccc} \mu & \alpha_{1} & \alpha_{2} & \alpha_{3} & \beta_{1} & \beta_{2} & \beta_{3} & \alpha \beta_{11} & \alpha \beta_{12} & \alpha \beta_{13} \end{array},\\ & \;\begin{array}{cccccccc} \;\;\;\;\alpha \beta_{21} & \alpha \beta_{22} & \alpha \beta_{23} & \alpha \beta_{31} & \alpha \beta_{32} & \alpha \beta_{33} & \delta_{1} & \delta_{2} \end{array}{]}^{\prime }, \end{array}$$

such that ***β*** is a 18 × 1 vector of fixed effects; that is, there are 18 elements in (A.2). Furthermore, **u** = [**u**′_*R*(*AB*)_ **u**′_*S*(*B*)_]′ can be similarly partitioned into a 18 × 1 vector of random effects, **u**_*R*(*A**B*)_, for replicates within inoculate by time and another 18 × 1 vector of random effects, **u**_*S*(*B*)_, for arrays within time; that is, there are a total of 18 biological replicates and 18 arrays in the study, each characterized by a random effect. Note that it is coincidence that the row dimensions of ***β***, **u**_*R*(*A**B*)_, and **u**_*S*(*B*)_ are all 18 for this particular example design.

Again, the distributional assumptions on the random and residual effects are specified the same as in the paper but now written in matrix algebra notation: **u**_*R*(*A**B*)_ ~ *N*(**0**_18×1_, **I**_18_ *σ*_*R*(*A**B*)_^2^), **u**_*S*(*B*)_ ~ *N*(**0**_18×1_, **I**_18_ *σ*_*S*(*B*)_^2^), and **e** ~ *N*(**0**_36×1_, *R* = **I**_36_ *σ*_*E*_^2^) with **0**_*t* × 1_ denoting a *t* × 1 vector of zeros and **I**_*t*_ denoting an identity matrix of dimension *t*. Reasonably assuming that **u**_*R*(*A**B*)_ and **u**_*S*(*B*)_ are pairwise independent of each other (i.e., biological sample effects are not influenced by array effects and vice versa), then the variance-covariance matrix **G** of **u** is a 36 × 36 diagonal matrix with the first 18 diagonal elements being *σ*_*R*(*A**B*)_^2^ and the remaining 18 diagonal elements being *σ*_*S*(*B*)_^2^. The GLS estimator, $\hat{\beta }$, of ***β*** can be written [22, 32] as

(A.3) β^=(X′V−1X)−X′V−1y,

with its variance-covariance matrix defined by

(A.4) var⁡(β^)=(X′V−1X)−,

such that (**X**′**V** ^−1^ **X**)^−^ denotes the generalized inverse of (**X**′**V** ^−1^ **X**).

Once the VC are estimated, they are substituted for the true unknown VC in **V** to produce V^ which are then used to provide the “estimated” GLS or EGLS $\tilde{\beta }$, of ***β***:

(A.5) β˜=(X′V^−1X)−X′V^−1y.

As noted in the text, typically β˜=β^ (i.e., EGLS = GLS) for balanced designs but not necessarily for unbalanced designs, such as those depicted in Figures 1 or 2.

It was previously noted in the paper that the mean difference *μ*_11._ − *μ*_21._ between inoculate *i* = 1 and *i* = 2 at time *j* = 1 as could be written as a function of the model effects in (1) as *α*_1_ − *α*_2_ + *α* *β*_11_ − *α* *β*_21_. Similarly, the mean difference *μ*_12._ − *μ*_12._ between time *j* = 1 and time *j* = 2 for inoculate *i* could be written as *β*_1_ − *β*_2_ + *α* *β*_11_ − *α* *β*_12_. These two contrasts written in matrix notation as **k**′_1_ ***β*** and **k**′_2_ ***β***, respectively, where

(A.6) $$\begin{array}{ll} k_{1}^{\prime } & =[\begin{array}{cccccccccccccccccc} 0 & 1 & -1 & 0 & 0 & 0 & 0 & 1 & 0 & 0 & -1 & 0 & 0 & 0 & 0 & 0 & 0 & 0 \end{array}], \end{array}$$

(A.7) $$\begin{array}{ll} k_{2}^{\prime } & =\left[\begin{array}{cccccccccccccccccc} 0 & 0 & 0 & 0 & 1 & -1 & 0 & 1 & -1 & 0 & 0 & 0 & 0 & 0 & 0 & 0 & 0 & 0 \end{array}\right] \end{array}$$

are *contrast vectors* whose coefficients align in order with the elements of ***β*** in (A.2). For example, note from (A.6) that the nonzero coefficients of 1, −1, 1, and −1 occur within the 2nd, 3rd, 8th, and 11th positions of **k**′_1_, respectively. When these coefficients are multiplied in the same order with the 2nd, 3rd, 8th, and 11th elements of ***β*** provided in (A.2), one gets (1)*α*_1_+(−1)*α*_2_ + (1)*α* *β*_11_ + (−1)*α* *β*_21_ which is indeed **k**′_1_ ***β*** = *α*_1_ − *α*_2_ + *α* *β*_11_ − *α* *β*_21_ as specified previously. The reader should be able to make a similar observation for **k**′_2_ ***β*** in considering how the nonzero elements of (A.7) align in position with elements of ***β*** in (A.2) to produce *β*_1_ − *β*_2_ + *α* *β*_11_ − *α* *β*_12_. In Figure 3, SAS PROC MIXED is used to provide the estimates, standard errors, and test statistics for these two contrasts. That is, note how all of the elements from (A.6) and (A.7) are completely reproduced in the *estimate* statements as “*k1 contrast*” and “*k2 contrast,*” respectively, in Figure 3.

Now, when the VC are known, these two contrasts can be estimated by their GLS, $k_{1}^{\prime }\hat{\beta },$ and $k_{2}^{\prime }\hat{\beta }$. Furthermore, using (A.4), the true standard errors of these two estimates can be determined as se(k1′β^)=k1′(X′V−1X)−k1 and se(k2′β^)=k2′(X′V−1X)−k2, respectively. However, as previously noted, the VC are generally not known but must be estimated from the data such that the two contrasts are typically estimated using $k_{1}^{\prime }\tilde{\beta }$ and $k_{2}^{\prime }\tilde{\beta }$ with approximate standard errors determined by se(k1′β˜)^=k1′(X′V^−1X)−k1 and se(k2′β˜)^=k2′(X′V^−1X)−k2. Using the REML estimates of VC as provided in the paper, the code from Figure 3 can be executed to provide $\hat{se\left(k_{1}^{\prime }\tilde{\beta }\right)}=0.2871$ whereas $\hat{se\left(k_{2}^{\prime }\tilde{\beta }\right)}=0.4085$ for ID_REF #30 by simply changing *method = type3* to *method = reml* and by deleting *ddfm = kr*. However, these standard errors are actually slightly understated since they do not take into account the uncertainty of the VC or V^ as an estimate of ****V**** as discussed by Kackar and Harville [38].

Kenward and Roger [39] derive a procedure to take this uncertainty into account and which is part of the SAS PROC MIXED implementation using the *ddfm=kr* option [35] as specified in Figure 3. Invoking this option raises the two standard errors accordingly, albeit very slightly, to $\hat{se\left(k_{1}^{\prime }\tilde{\beta }\right)}=0.2878$ and $\hat{se\left(k_{2}^{\prime }\tilde{\beta }\right)}=0.4088$. Furthermore, the *ddfm=kr* option invokes the procedure of Giesbrecht and Burns [40] to estimate the denominator degrees of freedom for EGLS inference. Using this option and REML, the estimated degrees of freedom for $k_{1}^{\prime }\tilde{\beta }$ is 5.28 whereas that for $k_{2}^{\prime }\tilde{\beta }$ is 17.0 as would be noted from executing the SAS code in Figure 3. The corresponding SAS output will furthermore include the *t*-test statistics for the two contrasts as t1=k1′β˜/se(k1′β˜)^=0.1328/0.2878=0.46 and t2=k2′β˜/se(k2′β˜)^=−0.3799/0.4088=−0.93. These statistics when compared to their Student *t* distributions with their respective estimated degrees of freedom, 5.28 and 17.0, lead to *P*-values of 0.66 and 0.37, respectively; that is, there is no evidence that either contrast is statistically significant.

*Contrast matrices* on ***β*** can be used to derive ANOVA-like *F* tests for the overall importance of various fixed effects using EGLS. Recall from the paper that the test for the main effects of inoculates can be written as a joint function of *v*_*A*_ = 2 contrasts *μ*_1.._ − *μ*_3.._ and *μ*_2.._ − *μ*_3.._, where *μ*_*i*.._ = (1/3)∑_*j*=1_^3^ *μ*_*i**j*._ with *μ* *_ij._* is defined as in (6). Thee two contrasts, labeled as (A1) and (A2) in the paper, can be jointly written together as a linear function **K**′_*A*_ ***β*** of the elements of ***β*** in (A.2), where

(A.8) $$K_{A}^{\prime }=\left[\begin{array}{cccccccccccccccccc} 0 & 1 & 0 & -1 & 0 & 0 & 0 & \frac{1}{3} & \frac{1}{3} & \frac{1}{3} & 0 & 0 & 0 & -\frac{1}{3} & -\frac{1}{3} & -\frac{1}{3} & 0 & 0\\ 0 & 0 & 1 & -1 & 0 & 0 & 0 & 0 & 0 & 0 & \frac{1}{3} & \frac{1}{3} & \frac{1}{3} & -\frac{1}{3} & -\frac{1}{3} & -\frac{1}{3} & 0 & 0 \end{array}\right].$$

For example, the first row of **K**′_*A*_ specifies the coefficients for testing *μ*_1.._ − *μ*_3.._ = (1/3)(*μ*_11._ + *μ*_12._ + *μ*_13._) − (1/3)(*μ*_31._ + *μ*_32._ + *μ*_33._) as a function of the elements of ***β*** using (6). In other words, matching up, in order, the first row of **K**′_*A*_ in (A.8) with the elements of ***β*** in (A.2), the corresponding contrast *μ*_1.._ − *μ*_3.._ can be rewritten as *α*_1_ − *α*_3_ + (1/3)*α* *β*_11_ + (1/3)*α* *β*_12_ + (1/3)*α* *β*_13_ − (1/3)*α* *β*_31_ − (1/3)*α* *β*_32_ − (1/3)*α* *β*_33_. Similarly, the second row of **K**′_*A*_ in (A.8) specifies the contrast coefficients for *μ*_2.._−*μ*_3.._ as a function of the elements of ***β***.

Recall that the main effects of times (Factor B) involves a joint test of *v*_*B*_ = 2 contrasts *μ*_.1._ − *μ*_.3._ and *μ*_.2._ − *μ*_.3._ labeled as (B1) and (B2) in the paper, where *μ*_.*j*._ = (1/3) ∑ _*i*=1_^3^ *μ*_*i**j*._. In terms of the elements of ***β*** in (A.2), these two contrasts are jointly specified as **K**′_*B*_ ***β*** with

(A.9) $$K_{B}^{\prime }=\left[\begin{array}{cccccccccccccccccc} 0 & 0 & 0 & 0 & 1 & 0 & -1 & \frac{1}{3} & 0 & -\frac{1}{3} & \frac{1}{3} & 0 & -\frac{1}{3} & \frac{1}{3} & 0 & -\frac{1}{3} & 0 & 0\\ 0 & 0 & 0 & 0 & 0 & 1 & -1 & 0 & \frac{1}{3} & -\frac{1}{3} & 0 & \frac{1}{3} & -\frac{1}{3} & 0 & \frac{1}{3} & -\frac{1}{3} & 0 & 0 \end{array}\right].$$

That is, (A.9) is another 2 × 18 contrast matrix, just like **K**′_*A*_, where the two rows of **K**′_*B*_ specify the coefficients for the contrasts *μ*_.1._ − *μ*_.3._ and *μ*_.2._ − *μ*_.3._, respectively, as a function of the elements of ***β*** in (6).

Recall that the interaction between the effects of inoculates and times was *v*_*A**B*_ = 4 numerator degrees of freedom test based on jointly testing four complementary and independent contrasts, suggesting that there are four rows that determine the corresponding contrast matrix. The complete interaction contrast can then be written as **K**′_*A**B*_ ***β***, where

(A.10) $$K_{AB}^{\prime }=\left[\begin{array}{cccccccccccccccccc} 0 & 0 & 0 & 0 & 0 & 0 & 0 & 1 & 0 & -1 & 0 & 0 & 0 & -1 & 0 & 1 & 0 & 0\\ 0 & 0 & 0 & 0 & 0 & 0 & 0 & 0 & 1 & -1 & 0 & 0 & 0 & 0 & -1 & 1 & 0 & 0\\ 0 & 0 & 0 & 0 & 0 & 0 & 0 & 0 & 0 & 0 & 1 & 0 & -1 & -1 & 0 & 1 & 0 & 0\\ 0 & 0 & 0 & 0 & 0 & 0 & 0 & 0 & 0 & 0 & 0 & 1 & -1 & 0 & -1 & 1 & 0 & 0 \end{array}\right].$$

Note that the 4 rows in (A.10) specify contrast coefficients on the model effects for each of the 4 constituent component hypotheses, (AB1), (AB2), (AB3), and (AB4) as defined in the paper, when aligned with the coefficients of ***β*** in (A.2). As a sidenote, the somewhat uninteresting contrast for dye effects could be written using a contrast vector **k**′_*D*_ (not shown) in order to test the overall mean difference between the two dyes.

The EGLS test statistic for testing the overall importance of any fixed effects term, say *X*, is specified as FX=β˜⁢ ′KX(X′V^−1X)−KX′β˜. Here *F*_*X*_ is distributed as an *F*-random variable under H_0_ : **K**′_*X*_ ***β*** = 0 with the numerator degrees of freedom being defined by the number of rows of the contrast matrix **K**′_*X*_ [27, 32, 35]. The denominator degrees of freedom for each contrast is dependent upon the design and can be determined based on that using classical ANOVA as in Table 1 or a multivariate extension of the Satterthwaite-based procedure from Giesbrecht and Burns [40] as proposed by Fai and Cornelius [41]; again this option is available as *ddfm=kr* using SAS PROC MIXED (Figure 3). The corresponding EGLS ANOVA output for ID_REF #30, based on either ANOVA or REML estimation of VC, is provided in Table 3.
